# Treatment of livestock with systemic insecticides for control of *Anopheles arabiensis* in western Kenya

**DOI:** 10.1186/s12936-015-0883-0

**Published:** 2015-09-17

**Authors:** Richard M. Poché, Dylan Burruss, Larisa Polyakova, David M. Poché, Rajesh B. Garlapati

**Affiliations:** Genesis Laboratories, P.O. Box 1195, Wellington, CO 80549 USA

**Keywords:** *Anopheles arabiensis*, Ivermectin, Eprinomectin, Fipronil, Endectocide, Malaria, Cattle, Kenya

## Abstract

**Background:**

Despite the implementation of vector control strategies, including insecticide-treated bed nets (ITN) and indoor residual spraying (IRS) in western Kenya, this area still experiences high level of malaria transmission. Novel vector control tools are required which target such vector species, such as *Anopheles arabiensis,* that feed outdoors and have minimal contact with ITNs and IRS.

**Methods:**

To address this need, ivermectin, eprinomectin, and fipronil were evaluated in Zebu cattle under semi-field conditions to evaluate the potential of these compounds to reduce the survival of blood feeding *An. arabiensis*. Over the course of four experiments, lactating cattle received doses of oral ivermectin at 0.1 or 0.2 mg/kg, oral eprinomectin at 0.2 or 0.5 mg/kg, topical eprinomectin at 0.5, 0.75, or 1.5 mg/kg, or oral fipronil at 0.25, 0.5, 1.0, or 1.5 mg/kg. On days 1, 3, 5, 7, 14, and 21 days post-treatment, cattle were exposed to *An. arabiensis*, and mosquito mortality post-blood feeding was monitored. For the analysis of survival data, the Kaplan–Meier estimator and Mantel–Haenszel test was used to contrast the treatment and control survival functions.

**Results:**

All three compounds significantly reduced the survival time of *An. arabiensis*. Twenty-one days post-treatment, mortality of mosquitoes fed on cattle dosed orally with 0.2 or 0.5 mg/kg eprinomectin, topically with eprinomectin at 0.5 mg/kg, or orally with either 1.0 or 1.5 mg/kg fipronil was still significantly higher than control mortality.

**Conclusions:**

These data demonstrate the effectiveness of three insecticidal compounds administered systemically to cattle for controlling the cattle-feeding mosquito *An. arabiensis*. Eprinomectin and fipronil provided the longest-lasting control. Such endectocidal treatments in cattle are a promising new strategy for control of residual, outdoor malaria transmission and could effectively augment current interventions which target more endophilic vector species.

**Electronic supplementary material:**

The online version of this article (doi:10.1186/s12936-015-0883-0) contains supplementary material, which is available to authorized users.

## Background

While great strides are being made towards reducing the worldwide malaria burden, malaria still caused an estimated 584,000 deaths in 2013, mostly among African children [1]. In Kenya, there are an estimated 6.7 million new clinical cases and 4000 deaths each year, and those living in western Kenya have an especially high risk of malaria [2]. Malaria prevalence is highest in Kenya in the lake endemic zone (38 %), is caused primarily by *Plasmodium falciparum* [3], and remains the most common cause of child morbidity in western Kenya [4]. In this region, *P. falciparum* is vectored by *Anopheles gambiae* sensu stricto *(s.s*.), *An. arabiensis*, and *Anopheles funestus s.s.* [5]. Given the behavioural diversity among these three vector species, a strategic investment must be made towards understanding their ecologies in order to design an effective, integrated management approach [6].

*Anopheles gambiaes.s*. and *An. funestuss.s.* are both highly anthropophilic, but *An. arabiensis* feeds readily on non-human vertebrates, particularly cattle [7–9]. Anthropophily in *An. arabiensis* also varies significantly, ranging from a high preference for human blood in West Africa to almost exclusive zoophily in Madagascar [7, 10–12]. In western Kenya, over half of the blood meals identified from *An. arabiensis* came from cattle, but a small proportion of *An. gambiaes.s*. also fed on cattle [13]. In areas where *An. arabiensis* is more anthropophagic, blood feeding still occurs predominately outdoors [14]. These behaviour traits make *An. arabiensis* less likely to encounter control strategies, which target endophagic and endophilic mosquitoes.

Zhou et al. [15] documented a resurgence of malaria parasite prevalence and malaria vectors in western Kenya despite increased usage of ITNs, which could be attributed to insecticide resistance and poor ITN coverage or usage. However, over the last 10 years, *An. gambiae s.s.* and *An. arabiensis* have also undergone changes in their relative abundance, likely influenced by the implementation of IRS and ITNs [13, 16]. While these strategies have led to the reduction of *An. gambiae s.s.* and *An. funestus s.s*., an unintentional consequence has been a proportionate increase in *An. arabiensis* [13]. Therefore, novel control strategies are needed for use in integrated malaria management programmes that target outdoor-feeding vectors not effectively controlled by ITNs and IRS.

One such approach is the use of “endectocides”, or treatment of a vertebrate host with a systemic insecticide that haematophagous arthropod vectors would become exposed to upon blood feeding. This host-targeted insecticide strategy for vector control has already been demonstrated effective in reducing sand fly vectors of visceral and cutaneous leishmaniasis [17–20], and flea vectors of plague [21, 22]. Targeting cattle, a frequent blood host of *An. arabiensis* [7–9, 12, 13], with a systemic insecticide may be an efficient approach to control this vector species. Foy et al. [23] discussed the application and potential impact of ivermectin and other endectocides on malaria control. Community-directed ivermectin treatment of humans is already main strategy for control of onchocerciasis [24], and has been successfully used in humans for malaria control as well [23, 25]. Many studies have demonstrated the lethal effect of ivermectin on mosquitoes after imbibing ivermectin-treated blood [26–30]. Eprinomectin is commercially used for control of endoparasites of livestock [31] and was demonstrated to be as effective as ivermectin at killing blood-feeding *An. gambiae s.s.* in the laboratory [30]. However, further investigation is needed to determine whether efficacy against mosquitoes is maintained in an in vivo system, and ascertain the duration of effectiveness. Fipronil is a broad spectrum insecticide which blocks the GABA-gated ion channels in the central nervous system [32]. Fipronil has been used to control ectoparasites on domestic animals [33], and as a pour-on or dip for cattle to control ticks [34, 35]. Mosquitoes are highly susceptible to fipronil during all life stages and by different routes of exposure [36–40]. However, field tests of fipronil as a systemic insecticide for mosquito control are currently lacking.

The long-term goal of the research is to create a product that can be utilized in an integrated malaria management programme, particularly to augment current control methodologies aimed at endophilic vectors by targeting more exophilic vectors with broader host utilization, such as *An. arabiensis*. To that end, this study examined the efficacy of ivermectin, eprinomectin and fipronil on the survivorship of adult *An. arabiensis.* The specific aim of this study was to determine the percent mortality of adult female *An. arabiensis* fed on cattle treated with different doses of ivermectin, eprinomectin, and fipronil, and determine the duration of this lethal effect post-treatment.

## Methods

### Study area

The study site was located 10 km west of Kisumu in the village of Kisian, Kenya (latitude −0.073220° and longitude 34.662974°).

### Cattle breed selection and cattle maintenance

All animal activities were reviewed and approved by the Institutional Animal Care and Use committees at Genesis Laboratories, Inc. and the Kenya Medical Research Institute (KEMRI). Lactating Zebu cattle (*Bos indicus*) were leased or purchased from markets or from private individuals. Cattle were transported to the study cattle shed located on the grounds of US Centers for Disease Control and Prevention and the Kenya Medical Research Institute (KEMRI), Kisian, Kenya. Transportation permits were provided by the department of veterinary services nearest to each purchase location. Test subjects were housed in individual stalls (1.5 × 3 m) within a covered cattle shed and were allowed periodic grazing in an outdoor pen during the 12-days acclimation period.

Upon arrival to the test facility each cow received an ear tag with a unique identification number and was inspected for general health. All test subjects were provided with clean tap water ad libitum and clean feed consisting of 8 kg of chopped Napier grass (*Pennisetum purpureum*) and 1.3 kg of dairy meal per day as directed by project veterinarians.

Cattle (test subjects) were maintained in a semi-controlled environment with adequate ventilation and natural light. Each test subject’s general health, and the daily temperature and relative humidity of the animal facility were documented by staff during the acclimation period and the test.

### Treatment randomization

A blocked randomization scheme by body weight was used to eliminate possible bias. Randomization was carried out using a random number generator service [41]. Each of the test subjects was assigned to either a control or treatment group. For each experiment, treatment groups which received doses of insecticide (test substance) consisted of three lactating Zebu cattle each, and the control group was allocated two lactating Zebu cattle. Precautions were taken to avoid animals contacting or grooming each other. The animals were housed individually in separate pens with a minimum distance to avoid contact between animals within and between treatment groups. Control animals were separated from the treatment animals.

### Administration of the test substance

Four experiments were conducted in order to evaluate multiple doses each of ivermectin, fipronil, and eprinomectin (Table 1). Experiment 1, conducted from 20 Dec 2012–22 Jan 2013, consisted or dosing cattle orally with eprinomectin at doses of 0.2 or 0.5 mg/kg, or topically with 0.5 mg/kg eprinomectin (Eprinex^®^). In experiment 2, conducted between 23 Jan 2013–26 Feb 2013, cattle were dosed orally with 0.1 or 0.2 mg/kg ivermectin, or topically with 0.75 mg/kg eprinomectin. In experiment 3, conducted from 25 Apr 2013–6 Jun 2013, cattle were dosed orally with either 1.0 or 1.5 mg/kg fipronil, or topically with 1.5 mg/kg eprinomectin. Experiment 4, conducted from 8 Aug 2013–22 Sept 2013, cattle received oral doses of fipronil at either 0.25 or 0.5 mg/kg. For each of these experiments, cows were randomized into 3 cows per treatment group and 2 cows per control for a total of 11 cows per experiment. Test substance quantity was calculated using weights recorded no more than 3 days prior to dosing. Topical and oral application methods of administering eprinomectin were chosen to assess efficacy and explore differences between application routes on mosquito survivorship.

**Table 1:** Listing of active ingredients, concentrations, route of administrations and total number of engorged mosquitoes used in the survival analysis per experiment

Experiment	Treatment group	Active ingredient	Concentration	Route of administration	Mosquito sample size
1	T0	Control	n/a	n/a	379
	T1	Eprinomectin	0.2 mg/kg	Oral	465
	T2	Eprinomectin	0.5 mg/kg	Oral	396
	T3	Eprinomectin	0.5 mg/kg	Topical	408
2	T0	Control	n/a	n/a	511
	T1	Ivermectin	0.1 mg/kg	Oral	475
	T2	Ivermectin	0.2 mg/kg	Oral	416
	T3	Eprinomectin	0.75 mg/kg	Topical	522
3	T0	Control	n/a	n/a	522
	T1	Eprinomectin	1.5 mg/kg	Topical	537
	T2	Fipronil	1.0 mg/kg	Oral	599
	T3	Fipronil	1.5 mg/kg	Oral	575
4	T0	Control	n/a	n/a	460
	T1	Fipronil	0.5 mg/kg	Oral	429
	T2	Fipronil	0.25 mg/kg	Oral	407

*Experiment 1* Cattle in treatment group one (T1) received an eprinomectin dose of 0.2 mg/kg orally, subjects in T2 received 0.5 mg/kg orally and subjects in T3 received 0.5 mg/kg topically. Because eprinomectin is not commercially available in oral formulations, crystalline eprinomectin was weighed in the laboratory and placed in a capsule for oral application. For T3, eprinomectin was applied topically using liquid Eprinex© (Merial Ltd., New Zealand) which was applied according to the manufacturer’s application directions. The manufacturer recommended application for Eprinex© pour-on commercial product is 1 ml/10 kg which would achieve a dosage of 0.5 mg eprinomectin/kg body weight.

*Experiment 2* Treatment group T1 received a 0.1 mg/kg ivermectin orally, subjects in T2 received 0.2 mg/kg ivermectin orally and subjects in T3 received 0.75 mg/kg eprinomectin topically. Ivermectin was administered orally using boluses; ivermectin tablets were weighed in the laboratory and placed in a capsule for oral application. Eprinomectin was applied topically using liquid Eprinex© applied according to the manufacturer’s application directions, but with a higher dose.

*Experiment 3* Treatment group T1 received a 1.5 mg/kg eprinomectin topically, subjects in T2 received 1.0 mg/kg fipronil orally and subjects in T3 received 1.5 mg/kg fipronil orally. Fipronil was administered orally using capsules. Technical grade fipronil was weighed in the laboratory and placed in a capsule for oral application. Eprinomectin was applied topically using liquid Eprinex©, applied as described above, but with a higher dose.

*Experiment 4* Treatment group T1 received a fipronil dose of 0.5 mg/kg orally while subjects in T2 received 0.25 mg/kg orally. Fipronil was weighed in a laboratory and placed in a capsule for oral application. For each experiment, the control group (T0) was left untreated.

### Animal subject performance

Clinical observations of test subjects were recorded daily by project staff during acclimation and experimentation phases of the study. In addition, a veterinarian conducted weekly health checks to more thoroughly examine test subject health. During application and experimentation periods, feed was weighed daily to assess the effects of test substances on the animals’ appetite. When spillage occurred, feed was returned to the appropriate container and weighed to the nearest 0.5 gram. Cattle weights were recorded on the final day of acclimation and weekly throughout the course of the study. Differences in appetite and body mass were compared by evaluating test subject weight means and standard deviations before and after treatment.

### Mosquito bioassays

All *An. arabiensis* used in this study were reared at the KEMRI/CDC, Kisian station, Kenya. Efficacy of each treatment was assessed by comparing survivorship of fully blood fed *An. arabiensis* at 1, 3, 5, 7, 14 and 21 days post treatment in experiments 1 and 2. While in experiment 3 mosquitoes were exposed at days 1, 7, 10, 14 and 21, in experiment 4 we exposed mosquitoes in days 1, 3, 5, 7, 14 and 21.

Prior to bioassays approximately 600 *An. arabiensis* adults were separated into an experimental cage and starved for 12 h. The day of application, 11–12 plastic capsules were filled with approximately 50 3–4 day-old female mosquitoes. Containers were modified round paper cartons that were 9.5 cm deep and 8.5 cm in diameter, covered with nylon netting material on one end to facilitate blood feeding. Containers with mosquitoes were transported in a cooler to and from the cattle shed.

The day before application all cows had a circular patch approximately 6 inches in diameter shaved on the ventral portion of the abdomen to expose skin and facilitate feeding. One container with *An. arabiensis* was applied to the shaved location of each test subject and secured by wrapping an ace bandage around the torso. One test subject in the control group received one capsules to ensure that the number of cartons applied to each group was equal. Containers were attached to test subjects for 30 min, and then carefully removed, and blood-fed females were counted. Unfed females were removed from the study.

Data were only analyzed for fully-engorged female mosquitoes. Blood fed females were placed into cages, provided with a 10 % sugar source ad libitum. For each group of mosquitoes in experiment 1 and 2, mortality was monitored at 3, 6 and 24 h post feeding and then daily for approximately 12 days thereafter. In experiment 3 and 4 we followed the same scheme but mortality was recorded daily for 9 days after the first 24 h.

### Statistical analysis

The statistical analysis of the survival data obtained from the control and treatment groups was conducted using the “survival” package [42] for the software R [43]. The package implements the Kaplan–Meier estimator, which is used to calculate the survival function of a random variable in time. A survival curve is the plot of the survival function representing the survivorship of the target population. The statistical difference between the control and the treatment was assessed using the Mantel–Haenszel test as implemented in the survival package. Values smaller than 0.05 represent a significant difference between the control and treatment group. The resulting survival functions were used to estimate the median survival time and 95 % confidence intervals for the estimate and the size of the effect of the active ingredient (Table 2).

**Table 2:** Median survival time and 95 % confidence interval per experiment

Hours/experiment	24 h (1 d)	72 h (3 d)	120 h (5 d)	168 h (7 d)	240 h (10 d)	336 h (14 d)	504 h (21 d)
Exp. 1 control	216 [168, 264]	144 [96, 192]	168 [144, 192]	96 [48, 216]	n/a	156 [96, 192]	120 [96, 168]
0.2 mg/Kg OE	192 [144, 264]	72 [72, 96]	120 [96, 168]	192 [168, 192]	n/a	24 [24, 96]	96 [96, 120]
0.5 mg/Kg OE	24 [24, 24]	48 [48, 72]	72 [72, 96]	168 [120, 216]	n/a	24 [24, 72]	96 [96, 96]
0.5 mg/Kg TE	48 [48, 72]	72 [48, 72]	48 [48, 72]	72 [72, 96]	n/a	48 [48, 72]	96 [96, 120]
Exp. 2 control	144 [120, 168]	72 [48, 120]	216 [168, 288]	240 [216, 264]	n/a	192 [168, 192]	120 [120, 120]
0.1 mg/Kg OI	96 [72, 96]	48 [48, 72]	132 [48, 264]	144 [96, 240]	n/a	192 [168, 216]	120 [120, 144]
0.2 mg/Kg OI	72 [72, 72]	60 [48, 72]	72 [72, 120]	144 [96, 168]	n/a	168 [144, 216]	120 [120, 120]
0.75 mg/Kg TE	48 [48, 48]	48 [24, 48]	48 [48, 48]	48 [48, 72]	n/a	168 [144, 192]	120 [120, 120]
Exp.3 control	n/a^1^ [−∞, ∞]	n/a	n/a	216 [216, 240]	168 [144, 168]	192 [192, 240]	n/a^1^ [240, ∞]
1.5 mg/Kg TE	24 [24, 24]	n/a	n/a	48 [24, 48]	72 [48, 96]	192 [192, 240]	n/a^1^ [216, ∞]
1.0 mg/Kg OF	24 [24, 24]	n/a	n/a	48 [48, 48]	72 [72, 96]	132 [72, 192]	144 [72, 240]
1.5 mg/Kg OF	24 [24, 24]	n/a	n/a	24 [24, 48]	48 [24, 48]	84 [48, 144]	72 [48, 168]
Exp. 4 control	144 [120, 144]	168 [168, 216]	n/a	192 [168, 240]	n/a	n/a^1^ [216, ∞]	240 [216, ∞]
0.25 mg/Kg OF	48 [48, 48]	120 [96, 144]	n/a	48 [48, 48]	n/a	204 [144, ∞]	216 [192, ∞]
0.5 mg/Kg OF	48 [48, 72]	120 [120, 144]	n/a	144 [144, 168]	n/a	216 [144, ∞]	n/a^1^ [216, ∞]

Values in square brackets represent the 95 % confidence intervals for the estimated median lethal time; n/a, time point not tested; n/a^1^, denotes estimate nor applicable because the survival function did not reach 0.5, therefore there is not a median value estimate; ∞, infinity, the survival function did not reach the corresponding to 95 % limit value

*h* hours, *d* days, *Exp.* experiment, *OE* oral eprinomectin, *TE* topical eprinomectin, *OI* oral ivermectin, *OF* oral fipronil

To compare the effect of time on the effectiveness of the test substance we did a post hoc analysis for the same concentration and delivery method (a single row on a table). For this, the significant level was adjusted using a Bonferroni correction (α/n, where α is the significance level set at 0.05 and n is the number of comparisons).

## Results

### Cattle observations, health, and performance

No adverse health effects arose in association with the treatments. For experiments 1–3, the test subject’s mean daily feed consumption did not differ between the acclimation and test periods. For experiment 4, test subjects’ mean daily feed consumption increased slightly from the acclimation (µ = 7.5 kg of hay/day, σ = 0.37) to the test period (µ = 7.8 kg of hay/day, σ = 0.02); *t* (15) = 2.13, p = 0.049. None of the cattle in any of the experiments experienced any large changes in their body mass over the course of the study.

### Mosquito bioassays

The data are presented in table form, with survival curves for all treatment groups and time points available in additional file 1. Table 2 shows the median survival time (and 95 % confidence intervals) with experiments separated by horizontal lines, each row correspond to a treatment (or control) in an experiment and each column is a time point when mosquitoes were challenged. Tables 3, 4, 5, 6 correspond to an experiment and each row shows the result for the active ingredient, concentration and delivery method. The columns represent the day post-exposure when mosquitoes were challenged against the test substance. The values shown are the p value of the comparison between a particular treatment and the control at a given day.

**Table 3:** Kaplan-Meier curve comparisons (p values) for oral and topical doses of eprinomectin (experiment 1)

Dose/days	1	3	5	7	14	21
0.2 mg/Kg oral	0.92	0.23	5.7 × 10^−4^*	0.11	3.26 × 10^−4^*	3.24 × 10^−4^*
0.5 mg/Kg oral	2.37 × 10^−37^*	3.74 × 10^−4^*	2.3 × 10^−6^*	0.12	0.09	4.43 × 10^−4^*
0.5 mg/Kg pour on	4.41 × 10^−7^*	8.82 × 10^−6^*	1.41 × 10^−7^*	0.148	0.001*	0.002*

* Comparison statistically different from the control at an adjusted α of 0.003

**Table 4:** Kaplan-Meier curve comparisons (p values) for oral doses of ivermectin and topical eprinomectin (experiment 2)

Dose/days	1	3	5	7	14	21
0.1 mg/Kg iver.	0.0003*	0.098	0.199	0.017	0.082	0.568
0.2 mg/Kg iver.	4.4 × 10^−16^*	0.004	0.0006*	3.77 × 10^−5^*	0.244	0.903
0.75 mg/Kg eprino.	0.0*	3.1 × 10^−11^*	4.1 × 10^−10^*	0.0*	0.161	0.858

* Comparison statistically different from the control at an adjusted α of 0.003

*Experiment 1* Mortality of mosquitoes fed on cattle dosed orally with 0.2 mg/kg eprinomectin was delayed during the days immediately following treatment. Survivorship of mosquitoes in this group was not significantly different from the controls until 5 days post-treatment, but then remained significant out to 21 days post-treatment (Table 3) with the exception of day 7. In contrast, mortality of mosquitoes fed on cattle dosed orally or topically with 0.5 mg/kg eprinomectin was significantly different from controls by 1 day post-treatment (Table 3). The 7-day time point post-treatment was an anomaly, a control replicate had a large mortality by 24 h.

*Experiment 2* At the lowest dose of ivermectin, 0.1 mg/kg, mosquito survivorship was marginally significantly different from the control at 1 day post-treatment, but then not significant (Table 4). For mosquitoes fed on cattle dosed with 0.2 mg/kg ivermectin, survivorship was significantly different from the controls at 1, 5 and 7, but not at 3, 14 and 21 days. For mosquitoes fed on cattle dosed topically with 0.75 mg/kg eprinomectin, survivorship was significantly different from the controls from 1 to 7 days, but not at 14 or 21 days (Table 4).

*Experiment 3* For mosquitoes fed on cattle dosed topically with 1.5 mg/kg eprinomectin, survivorship was significantly different from the control out to 10 days, but not at 14 or 21 days (Table 5). For both doses of fipronil, mosquito survivorship was significantly different from the control at all time points out to 21 days (Table 5).

**Table 5:** Kaplan-Meier curve comparisons (p values) for oral doses of fipronil and topical eprinomectin (experiment 3)

Dose/days	1	7	10	14	21
1.5 mg/Kg eprino.	0.0*	0.0*	5.3 × 10^−11^*	0.734	0.351
1.0 mg/Kg fipro.	0.0*	0.0*	0.0*	0.006*	0.001*
1.5 mg/Kg fipro.	0.0*	0.0*	0.0*	8.32 × 10^−11^*	5.52 × 10^−12^*

* Comparison statistically different from the control at an adjusted α of 0.003

*Experiment 4* For both doses of fipronil, mosquito survivorship was significantly different from the control out to 7 days (Table 6). Mosquito survivorship was not significantly different from the control for either dose at 14 and 21 days (Table 6).

**Table 6:** Kaplan-Meier curve comparisons (p values) for oral doses of fipronil (experiment 4)

Dose/days	1	3	7	14	21
0.25 mg/Kg fipro.	0.0*	5.0 × 10^−6^*	5.5 × 10^−14^*	0.10	0.768
0.5 mg/Kg fipro.	0.0*	5.7 × 10^−8^*	6.8 × 10^−5^*	0.10	0.171

* Comparison statistically different from the control at an adjusted α of 0.005

## Discussion

This study evaluated the endectocidal activity of three compounds in cattle against *An. arabiensis* mosquitoes in a semi-field environment. Positive results were achieved with each test substance, but with varying degrees of efficacy depending on dose and route of administration.

### Ivermectin

Ivermectin mass drug administration (MDA) to humans for onchocerciasis control has been demonstrated to also reduce malaria parasite transmission by affecting mosquito survivorship, vector competence, re-feeding rates, and parity [25, 29, 44, 45]. When administered to humans during MDA campaigns, the standard oral dose of ivermectin is 150 µg/kg. While the aforementioned studies and others have well-characterized the use of ivermectin as a human endectocide for malaria vector control, this study was one of the first to evaluate the use of ivermectin in cattle for control of *An. arabiensis*.

Fritz et al. [26] evaluated a commercially-available injectable formulation of ivermectin in cattle, and found that most (90 %) of the *An. gambiaes.s.* that fed on the ivermectin-treated cattle within two weeks of treatment failed to survive more than 10 days post-blood meal. Further, no eggs were deposited by *An. gambiaes.s*. that fed on ivermectin-treated cattle within 10 days of treatment [26]. These results are promising, however injectable formulations are difficult to administer and require veterinary expertise. In that light, the current study evaluated two oral doses of ivermectin. Of these oral formulations, the higher of the two doses (0.2 mg ivermectin/kg) achieved significant results out to 7 days (168 h) post-treatment. This result is also consistent with the described pharmacokinetics of this compound. Ivermectin has an elimination half-life of 32–178 h when administered intravenously, depending on species [46]. Day 3, in the ivermectin experiment, had a control replicate with large mortality (38 %) by 24 h. If the control replicate is removed, the median survival time in control is increased to 96 h; 95 % confidence interval [48, 120 h] (Table 2). The 0.1 mg/Kg ivermectin treatment remains insignificant, while the 0.2 mg/Kg of ivermectin treatment becomes significant (p = 0.001).

A significant effect on mosquito survivorship for approximately 1 week also corroborates the results obtained by Alout et al. [25], whereby a 33.9 % reduction in survivorship of *An. gambiaes.s.* was observed for 7 days following a MDA in humans. While this effect on mosquito survival was brief, a significant reduction in mosquito parity rates was observed for more than 2 weeks after the MDA [25]. Additionally, sporozoite rates were reduced by 77.5 % for 15 days [25]. Kobylinski et al. [45] similarly also observed a 79 % reduction in sporozoite-positive *An. gambiaes.s*. for over 2 weeks following MDA. Therefore, ivermectin treatments in cattle may similarly impact the vectorial capacity of *An. arabiensis* in a field situation, and warrant further field investigation.

### Eprinomectin

Eprinomectin has established utility in the agricultural industry as an effective means to control endoparasite loads in cattle [31], with the additional health benefits of increasing cattle weight gain and milk production [31, 47]. However, eprinomectin has not been widely used for public health purposes. Butters et al. [30] evaluated eprinomectin alongside several other active ingredients in the laboratory for control of *An. gambiae* s.s. and found it had a similar LC_50_ to ivermectin. Fritz et al. [48] also evaluated eprinomectin and ivermectin in the laboratory against *An. arabiensis* and found both compounds to be effective at killing mosquitoes at concentrations under 10 parts per billion. However, no studies to date have evaluated eprinomectin under field conditions for control of anopheline malaria vectors.

In this study two oral (0.2 and 0.5 mg/kg) and three topical (0.5, 0.75, and 1.5 mg/kg) doses of eprinomectin were evaluated. Of the oral formulations, the lower dose demonstrated a delayed effectiveness, with a significant effect on mosquito mortality at time points from 5 to 21 days post-treatment, but not at 7 days. In contrast, the 0.5 mg/kg dose had a significant effect on mosquito mortality up to 5 days post-treatment and again at 21 days, but not at 7 or 14 days. Of the topical (pour-on) formulations, significant effects were observed immediately for all three doses (1 day post-treatment) (Tables 3, 4, 5), however, the lowest treatment (0.5 mg/kg) resulted in significant mosquito mortality for 21 days with the exception of day 7 (Table 3), however as previously mentioned, day 7 of experiment 1 had large mortality in a control replicate (12/17 dead by 24 h). Removing this replicate increases the median survival time in the control from 96 h (95 % confidence interval 48, 116 h) to 192 h (95 % confidence interval 120, 264 h). As a result the topical 0.5 mg/kg eprinomectin treatment becomes statistically significant (p = 5.9 × 10^−4^).

For reasons unclear, the eprinomectin higher doses were effective for shorter periods of time (7 days for 0.75 mg/kg for and 10 days for 1.5 mg/kg) than the lower doses (21 days for 0.5 and 0.2 mg/Kg). The long-lasting low-dose effect of eprinomectin are unexpected and despite a large sample size (Table 1) and low variability (Table 2) further experimentation will be necessary to confirm these results. Oral formulations of eprinomectin are currently not commercially available, but should be further developed for study due to the potential for low concentrations to have a significant killing effect on mosquitoes (Table 3).

The same dose and route of administration was assessed for eprinomectin and ivermectin, although in separate experiments. When comparing 0.2 mg/kg oral ivermectin (Table 4) and 0.2 mg/kg oral eprinomectin (Table 3), ivermectin was immediately effective with significant mosquito mortality out to 7 days post-treatment, whereas the effectiveness of eprinomectin was delayed, but lasted out to 21 days. Laboratory studies comparing eprinomectin and ivermectin have also demonstrated comparable effectiveness of both compounds but with slightly different pharmacokinetics. Butters et al. [30] reported significant knockdown of *An. gambiaes.s.* with both ivermectin and eprinomectin, however, the knockdown effect of eprinomectin was within the first hour following the blood meal whereas the knockdown effect for ivermectin was not apparent until 24 h after the blood meal [30]. The discrepancy between our results and those of Butters et al. [30] may relate to the difference in pharmacokinetics of these compounds under laboratory and in vivo conditions. In the laboratory where mosquitoes were exposed to blood spiked with the active ingredient, the results obtained would related directly to the activity of the compound itself in the absence of any metabolites or conditions associated with feeding on treated cattle. Ivermectin and eprinomectin have similar plasma kinetics and mean residence time (the amount of time one molecular stays in the organism) when administered to mice intravenously and orally, however with some variation in the rate and mechanism of drug elimination [49]. With the information available, it is also difficult to compare the concentration of active ingredient mosquitoes would have been exposed to at corresponding time points between these publications, or to know the relative contribution made by mosquito genetics, since Butters et al. [30] utilized *An. gambiaes.s.* G3 strain, and this study used *An. arabiensis* sourced in Kenya. Further study is warranted to ascertain the nature of the delayed knock-down effect observed in *An. arabiensis* when exposed to eprinomectin circulating in cattle treated orally. More work is also needed to assess the complementary uses of these compounds in the field. Since eprinomectin is not approved for human use as is the case ivermectin, endectocidal treatments in cattle with eprinomectin may be a complementary approach to the use of ivermectin in people when both *An. gambiaes.s*. and *An. arabiensis* are present.

### Fipronil

Cattle were dosed orally with four different doses of fipronil over the course of two experiments. Fipronil dosing significantly reduced mosquito survivorship for at least 21 days when cattle were administered either 1.0 or 1.5 mg/kg, and for at least 7 days at the lower doses of 0.25 and 0.5 mg/kg. A significant effect may have occurred at the 14-day time point for the 0.25 and 0.5 mg/kg concentrations; however these data could not be analysed due to unexplained mortality in the control groups. Poché et al. [19] also tested fipronil as an endectocide in cattle, although for control of sand fly vectors of visceral leishmaniasis in India. In that study, four oral dose levels were evaluated: 0.5, 1.0, 2.0, and 4.0 mg/kg. Between 20 % (0.5 mg/kg) and 100 % (4.0 mg/kg) mortality was observed in adult *Phlebotomus argentipes* sand flies fed on treated cattle 21 days post-treatment with fipronil [19]. At the 1.0 mg/kg dose level in both studies, control of adult sand flies [19] and mosquitoes (this study) was significantly different from the controls at 21 days post treatment. This long-lasting efficacy makes fipronil a strong candidate for future malaria control field studies.

The use of fipronil as a public health endectocide has already been extensively evaluated for control of adult and larval sand fly vectors of leishmaniasis. In India, fipronil treatment of two rodent species resulted in 100 % mortality of *P. argentipes* larvae following consumption of treated feces [18]. In that same study, 100 % mortality of blood-feeding adult *P. argentipes* was also achieved when sand flies were allowed to feed on rodents up to 20 days post-treatment [18]. In Tunisia, Derbali et al. [20] reported that fipronil – treated baits consumed by the desert’s jird (*Meriones shawi*) had a systemic effect on the survival of *Phlebotomus papatasi* after blood meal acquisition, as well as a feed-through effect on the survival of larval *P. papatasi* after consumption of feces. And as mentioned above, treatment of cattle with fipronil also successfully controlled adult and larval *P. argentipes* for 21 days [19]. Lopes et al. [35] also used fipronil treatment of cattle to control ivermectin-resistant cattle ticks, *Rhipicephalus (Boophilus) microplus*. The topically administered fipronil formulation (1 mg/kg) achieved efficacy values greater than 95 % from 3 to 28 days after treatment. On 35, 42 and 49 days post-treatment, efficacy values were 94, 78 and 61 %, respectively [35]. The application of fipronil as a cattle endectocide for malaria control is a natural extension of these studies.

## Conclusions

Ivermectin, eprinomectin, and fipronil each show promising potential as endectocides administered to cattle for lowering the survival rate of *An. arabiensis* mosquitoes, and hence reducing malaria transmission rates. Mosquito mortality was significantly higher than control mortality as long as 21 days post-treatment after mosquitoes fed on cattle dosed orally with 0.2 or 0.5 mg/kg eprinomectin, topically with eprinomectin at 0.5 mg/kg, or orally with either 1.0 or 1.5 mg/kg fipronil. Other components of vectorial capacity were not evaluated, and would be valuable to incorporate into future studies. Endectocidal treatments in cattle are a promising new strategy for control of residual, outdoor malaria transmission driven by vectors that feed on cattle, and could effectively augment current interventions which target more endophilic vector species.

## Additional file

**Additional file 1.** Survival curves for all treatment groups and time points.
