# Supplementary material for: Treatment of livestock with systemic insecticides for control of Anopheles arabiensis in western Kenya
Source: Malar J. 2015 Sep 17;14:351. doi: 10.1186/s12936-015-0883-0 (PMC4574316; doi:10.1186/s12936-015-0883-0)
Supplement: Supplementary file 1 — Additional file 1. Survival curves for all treatment groups and time points. [file 12936_2015_883_MOESM1_ESM.docx]

**Experiment 1, 1-day:** Survival of *An. arabiensis* at 24h post-treatment. T0: control; T1: eprinomectin 0.2 mg/kg orally; T2: eprinomectin 0.5 mg/kg orally; T3: eprinomectin 0.5 mg/kg topically. Time presented in hours; curves bound by 95% confidence intervals.

**Experiment 1, 3-day:** Survival of *An. arabiensis* at 72h post-treatment. T0: control; T1: eprinomectin 0.2 mg/kg orally; T2: eprinomectin 0.5 mg/kg orally; T3: eprinomectin 0.5 mg/kg topically. Time presented in hours; curves bound by 95% confidence intervals.

**Experiment 1, 5-day:** Survival of *An. arabiensis* at 120h post-treatment. T0: control; T1: eprinomectin 0.2 mg/kg orally; T2: eprinomectin 0.5 mg/kg orally; T3: eprinomectin 0.5 mg/kg topically. Time presented in hours; curves bound by 95% confidence intervals.

**Experiment 1, 7-day:** Survival of *An. arabiensis* at 168h post-treatment. T0: control; T1: eprinomectin 0.2 mg/kg orally; T2: eprinomectin 0.5 mg/kg orally; T3: eprinomectin 0.5 mg/kg topically. Time presented in hours; curves bound by 95% confidence intervals.


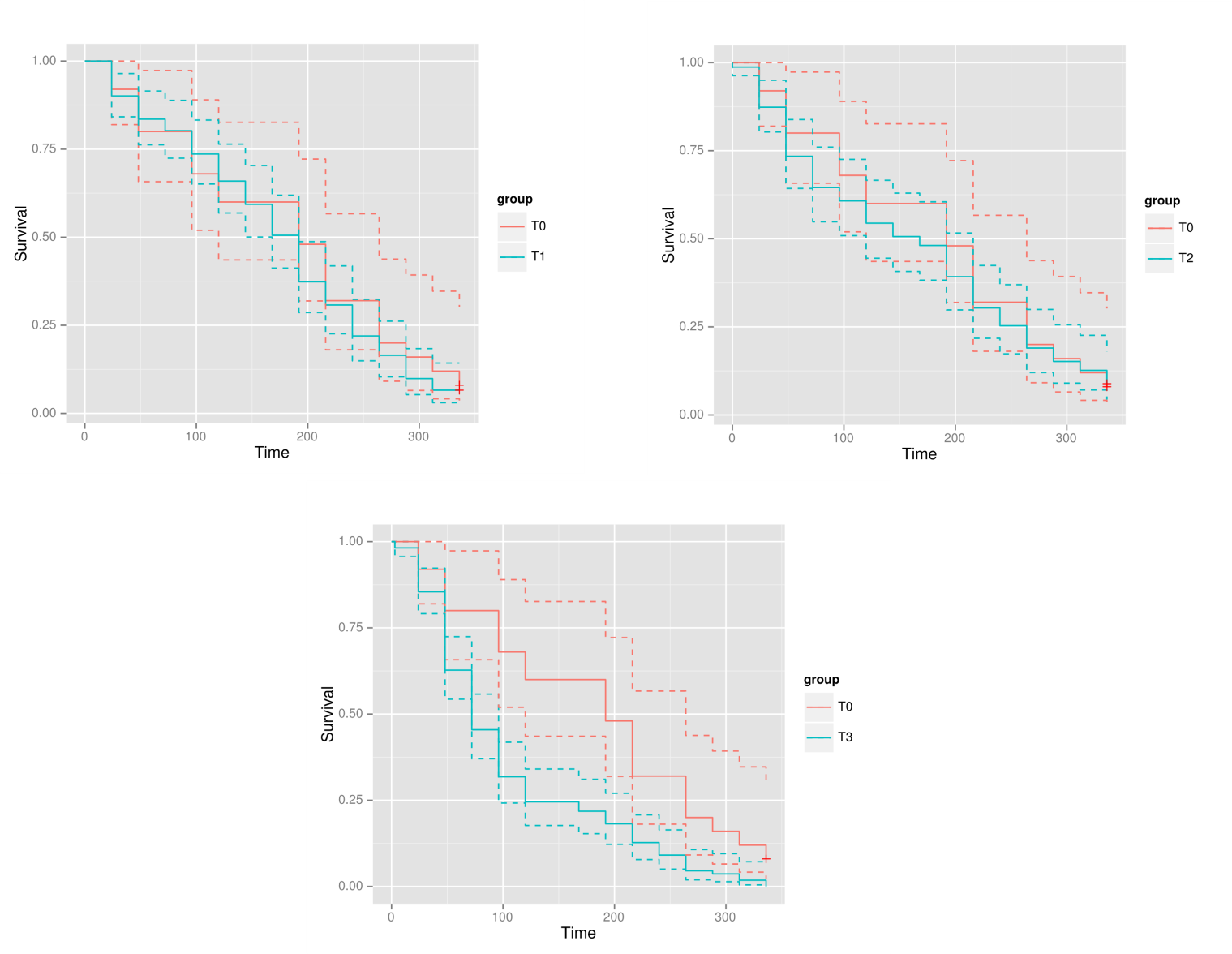


**Experiment 1, 14-day:** Survival of *An. arabiensis* at 336h post-treatment. T0: control; T1: eprinomectin 0.2 mg/kg orally; T2: eprinomectin 0.5 mg/kg orally; T3: eprinomectin 0.5 mg/kg topically. Time presented in hours; curves bound by 95% confidence intervals.

**Experiment 1, 21-day:** Survival of *An. arabiensis* at 504h post-treatment. T0: control; T1: eprinomectin 0.2 mg/kg orally; T2: eprinomectin 0.5 mg/kg orally; T3: eprinomectin 0.5 mg/kg topically. Time presented in hours; curves bound by 95% confidence intervals.

**Experiment 2, 1-day:** Survival of *An. arabiensis* at 24h post-treatment. T0: control; T1: ivermectin 0.1mg/kg orally; T2: ivermectin 0.2mg/kg orally; T3: eprinomectin 0.75mg/kg topically. Time presented in hours; curves bound by 95% confidence intervals.

**Experiment 2, 3-day:** Survival of *An. arabiensis* at 72h post-treatment. T0: control; T1: ivermectin 0.1mg/kg orally; T2: ivermectin 0.2mg/kg orally; T3: eprinomectin 0.75mg/kg topically. Time presented in hours; curves bound by 95% confidence intervals.

**Experiment 2, 5-day:** Survival of *An. arabiensis* at 120h post-treatment. T0: control; T1: ivermectin 0.1mg/kg orally; T2: ivermectin 0.2mg/kg orally; T3: eprinomectin 0.75mg/kg topically. Time presented in hours; curves bound by 95% confidence intervals.

**Experiment 2, 7-day:** Survival of *An. arabiensis* at 168h post-treatment. T0: control; T1: ivermectin 0.1mg/kg orally; T2: ivermectin 0.2mg/kg orally; T3: eprinomectin 0.75mg/kg topically. Time presented in hours; curves bound by 95% confidence intervals.

**Experiment 2, 14-day:** Survival of *An. arabiensis* at 336h post-treatment. T0: control; T1: ivermectin 0.1mg/kg orally; T2: ivermectin 0.2mg/kg orally; T3: eprinomectin 0.75mg/kg topically. Time presented in hours; curves bound by 95% confidence intervals.

**Experiment 2, 21-day:** Survival of *An. arabiensis* at 504h post-treatment. T0: control; T1: ivermectin 0.1mg/kg orally; T2: ivermectin 0.2mg/kg orally; T3: eprinomectin 0.75mg/kg topically. Time presented in hours; curves bound by 95% confidence intervals.

**Experiment 3, 1-day:** Survival of *An. arabiensis* at 24h post-treatment. T0: control; T1: eprinomectin 1.5 mg/kg topically; T2: fipronil 1.0 mg/kg orally; T3: fipronil 1.5mg/kg orally. Time presented in hours; curves bound by 95% confidence intervals.

**Experiment 3, 7-day:** Survival of *An. arabiensis* at 168h post-treatment. T0: control; T1: eprinomectin 1.5 mg/kg topically; T2: fipronil 1.0 mg/kg orally; T3: fipronil 1.5mg/kg orally. Time presented in hours; curves bound by 95% confidence intervals.

**Experiment 3, 10-day:** Survival of *An. arabiensis* at 240h post-treatment. T0: control; T1: eprinomectin 1.5 mg/kg topically; T2: fipronil 1.0 mg/kg orally; T3: fipronil 1.5mg/kg orally. Time presented in hours; curves bound by 95% confidence intervals.

**Experiment 3, 14-day:** Survival of *An. arabiensis* at 336h post-treatment. T0: control; T1: eprinomectin 1.5 mg/kg topically; T2: fipronil 1.0 mg/kg orally; T3: fipronil 1.5mg/kg orally. Time presented in hours; curves bound by 95% confidence intervals.

**Experiment 3, 21-day:** Survival of *An. arabiensis* at 504h post-treatment. T0: control; T1: eprinomectin 1.5 mg/kg topically; T2: fipronil 1.0 mg/kg orally; T3: fipronil 1.5mg/kg orally. Time presented in hours; curves bound by 95% confidence intervals.

**Experiment 3, 28-day:** Survival of *An. arabiensis* at 672h post-treatment. T0: control; T1: eprinomectin 1.5 mg/kg topically; T2: fipronil 1.0 mg/kg orally; T3: fipronil 1.5mg/kg orally. Time presented in hours; curves bound by 95% confidence intervals.

**Experiment 4, 1-day:** Survival of *An. arabiensis* at 24h post-treatment. T0: control; T1: fipronil 0.5mg/kg orally; T2: fipronil 0.25 mg/kg orally. Time presented in hours; curves bound by 95% confidence intervals.

**Experiment 4, 3-day:** Survival of *An. arabiensis* at 72h post-treatment. T0: control; T1: fipronil 0.5mg/kg orally; T2: fipronil 0.25 mg/kg orally. Time presented in hours; curves bound by 95% confidence intervals.

**Experiment 4, 5-day:** Survival of *An. arabiensis* at 168h post-treatment. T0: control; T1: fipronil 0.5mg/kg orally; T2: fipronil 0.25 mg/kg orally. Time presented in hours; curves bound by 95% confidence intervals.

**Experiment 4, 14-day:** Survival of *An. arabiensis* at 336h post-treatment. T0: control; T1: fipronil 0.5mg/kg orally; T2: fipronil 0.25 mg/kg orally. Time presented in hours; curves bound by 95% confidence intervals.

**Experiment 4, 21-day:** Survival of *An. arabiensis* at 504h post-treatment. T0: control; T1: fipronil 0.5mg/kg orally; T2: fipronil 0.25 mg/kg orally. Time presented in hours; curves bound by 95% confidence intervals.
